# Designing a Computer-Vision Application: A Case Study for Hand-Hygiene Assessment in an Open-Room Environment

**DOI:** 10.3390/jimaging7090170

**Published:** 2021-08-30

**Authors:** Chengzhang Zhong, Amy R. Reibman, Hansel A. Mina, Amanda J. Deering

**Affiliations:** 1 The Elmore Family School of Electrical and Computer Engineering, Purdue University, West Lafayette, IN 47907, USA; zhongc@purdue.edu; 2 Department of Food Science, Purdue University, West Lafayette, IN 47907, USA; hminacor@purdue.edu (H.A.M.); adeering@purdue.edu (A.J.D.)

**Keywords:** activity recognition, deep learning, domain adaptation

## Abstract

Hand-hygiene is a critical component for safe food handling. In this paper, we apply an iterative engineering process to design a hand-hygiene action detection system to improve food-handling safety. We demonstrate the feasibility of a baseline RGB-only convolutional neural network (CNN) in the restricted case of a single scenario; however, since this baseline system performs poorly across scenarios, we also demonstrate the application of two methods to explore potential reasons for its poor performance. This leads to the development of our hierarchical system that incorporates a variety of modalities (RGB, optical flow, hand masks, and human skeleton joints) for recognizing subsets of hand-hygiene actions. Using hand-washing video recorded from several locations in a commercial kitchen, we demonstrate the effectiveness of our system for detecting hand hygiene actions in untrimmed videos. In addition, we discuss recommendations for designing a computer vision system for a real application.

## 1. Introduction

This paper presents an exploration to build a video-analytics system for hand-hygiene assessment in an open-room environment. While hand hygiene is important in hospital and health care settings as well as in our daily life, here we focus on hand hygiene for safe food handling, where it is a critical component [1] to ensure a healthy food supply. Traditionally, assessing the quality of handwashing in a food processing facility would require the food business owner to hire a food expert to conduct an audit, which is expensive. With the recent growth of automated video processing, or video analytics, using a video analytic system to replace human observations becomes possible. Compared to the cost of hiring a person, an automated system only requires cameras, a personal computer, and effective algorithms. Such a system could accomplish a significant part of a food handling audit much more inexpensively.

In general, hand hygiene can be decomposed into a list of steps that need to be accomplished, in many cases in a prescribed order or for a sufficiently long duration [2]. From the perspective of video analytics, hand-hygiene quality can be assessed using either action recognitionor action detection. Each step in the hand-hygiene procedure can be considered to be an individual action class. For action recognition, the task is to recognize one single action that occurs in a trimmed video clip. In contrast, action detection processes untrimmed videos that contain more than one action, and the task is to localize all actions as well as identify the class, or category, of each action.

Our previous work [3] presented a two-stage multi-camera system for hand-hygiene quality assessment. In it, we focused on hand-hygiene assessment in a constrained restroom environment using both stationary and body-mounted cameras. In this paper, we extend this work to consider an open-room environment with a single side-view stationary camera. We use the term “open-room environment” to contrast these environments with both the much smaller restrooms of our previous work and the top-down sink-only views of [4,5,6]. In the current open-room environment, the distances between the camera and the sink are much longer, and there are potentially many more distracting objects. As a result, more video-processing challenges exist, so more sophisticated methods are necessary. In particular, today’s computer vision techniques rely heavily on the availability of data that have been painstakingly labeled, and yet the methods are known to be brittle when they are applied in a scenario where the data have different characteristics. Overcoming this brittleness for our hand-hygiene application will be the focus of this paper. We anticipate this paper will be of interest to practitioners of computer vision who want to build a practical computer vision system that operates in a real-world environment.

The system we describe in this paper is motivated by several design goals. First, similar to [3,7], we need to detect when, and possibly for how long, the basic hand-hygiene actions occur. Second, the system must perform well in an open-room environment. In particular, it must work effectively across multiple scenarios, in multiple rooms, or with different camera angles. Finally, due to the limited availability of data, the system must be designed to avoid over-fitting. In the course of the design, we also need to define what actions are to be detected and decide which computer vision methods to leverage, including which system architectures to apply with what inputs and specific classification problems to answer. All these steps are necessary components of the design process for any real-world video analytics system.

The open-room environment is especially challenging for an effective video analytics system. Because of the variations of possible open-room environments, the data that can be gathered will never be comprehensive. It is impossible to sample all potential open-room environments to train a machine-learning method that operates well in all of them. Variations include the camera angle, height, and distance from the sink; the different appearances, heights, and locations of required objects like the soap, sink, and faucets; and the placement and presence of extraneous objects that are not necessary for hand-hygiene. Moreover, the method must be robust to different people, since no two people are likely to perform the same actions in the same way.

To begin our design of an effective action detection system for the open-room environment, we design a baseline action recognition system for a single scenario: a given room with a given camera placement. This design requires a sufficient amount of annotated video data from that scenario, and assumes that the distribution of data used for the training and testing stages are similar.

In addition, we extend our design for action recognition by presenting a hierarchical system with multi-modal inputs. Our motivation in this new design is to create a model trained from data in one scenario that can also perform well across scenarios. In real-world situations, we may not be able to have guaranteed access to the environment in which we would like to deploy a system prior to the actual deployment. This may be because we do not have permission to collect video data in that target scenario or we may be uncertain about where that target scenario may be. This implies that the data from the target scenario are either limited, unlabeled, or completely missing. For this, we apply three strategies motivated by the literature and described in more detail in Section 2.3 to construct a system using data from one scenario but deploy it in a target scenario, a concept known as *transfer learning*.

This work extends our previous work [3,7] in three main ways. First, we extend hand-hygiene action recognition from a single restroom-sink scenario into food-handling laboratory scenarios with a variety of camera views and background layouts. The extension allows us to assess the robustness our hand-hygiene method under different situations. Second, we explore the gap between hand-hygiene action recognition in the same scenario and across scenarios, and consider the use of multiple modalities to address the challenges. Third, we propose a hierarchical system that combines multi-modal inputs to recognize hand-hygiene actions across scenarios.

Our contributions in this paper are as follows:

1. A multi-modality framework to recognize cross scenario hand-hygiene actions in untrimmed video sequences. This hierarchical system incorporates a variety of modalities (RGB, optical flow, hand masks, and human skeleton joints) for recognizing certain subsets of hand-hygiene actions. Combined, these modalities perform effective hand hygiene assessment across multiple scenarios.
2. A comparison and evaluation of the performance of a baseline spatial-only deep learning model and a spatio-temporal model on the same scenario hand-hygiene situation to process both trimmed and untrimmed hand-hygiene videos. We demonstrate that the spatial-only model has equally good performance as the spatio–temporal model for hand-hygiene recognition in the same scenario.
3. Demonstration of two methods that explore potential reasons why our baseline RGB-only model performs poorly across scenarios. These indicate that a primary reason models designed for one scenario do not transfer well into a second scenario is that the model may have learned to focus on irrelevant objects.

In Section 2 we review previous approaches for image and video processing for hand-hygiene assessment and provide background on recent relevant approaches in computer vision and video analytics. Section 3 describes the engineering process we performed during the course of this research. Section 4 describes our video data collection using multiple side-view cameras in a commercial kitchen. Our baseline system and its performance is presented in Section 5, and Section 6 presents two additional experiments to explore why our baseline system performs well in a same-scenario situation but not in a cross-scenario situation. Section 7 presents our hierarchical system and its subsystems, and performance results are presented in Section 8. We discuss limitations of our system as well as advice for others implementing real-world video analytics applications in Section 9, and provide concluding thoughts in Section 10.

## 2. Background and Motivation

In this section, we describe both previous systems that have been developed to assess and assist hand-hygiene activities using image and video processing (Section 2.1); we also describe background information about video analytics and computer vision methods for action recognition, action detection, and domain adaptation (Section 2.2). Section 2.3 describes how the previous work in these areas motivates the design of our hand-hygiene system. In particular, this section describes the three strategies we apply here to create an effective cross-scenario hand-hygiene system.

### 2.1. Applications for a Hand-Hygiene Assessment System

Hand hygiene plays a critical role in reducing contamination in our food supply and in reducing disease transmission. Several image- and video-based systems have been developed to assess or assist hand hygiene for healthcare workers [5,6,8,9,10,11,12] and people with dementia [4,13,14,15]. In [8,9], image processing reports when hospital staff are present at a hand-washing station. To assess the effectiveness of hand-washing after the fact, images of the hands using both visible and ultraviolet light have been processed [10,11,12]. Six handwashing poses are classified using multiple SVMs in [6], by segmenting hands and arms and analyzing their motion. Robustness to lighting conditions is obtained by combining skin tone and motion during hand segmentation. A real-time and improved version of this system using linear discriminant analysis is presented in [5]. The COACH system (Cognitive Orthosis for Assisting aCtivities in the Home) is an interactive system that prompts patients with dementia to perform correct hand-hygiene when they are unable to perform it on their own [4]. The COACH system tracks hands and towels using particle filters [13], and detects waterflow by combining both audio and video processing [14]. Automated video analysis has also been explored as a means to assess cognitive behavior in older adults, where the key indicators considered were occupancy of different regions of the sink, and the path tortuosity of the motion trajectory of the hands [15].

In this paper, as in our previous work [3,7], we consider a different application of hand-hygiene assessment: reducing pathogens in food-handling facilities. It is estimated that each year over 9 million people suffer from a food-bourne illness in the United States alone [16]. Evidence indicates that the majority of food contamination is caused by inappropriate food manufacturing practices involving workers with poor food handling skills [1]. Effective hand-hygiene reduces the likelihood that food handlers transfer pathogenic microorganisms from their hands to food products [17]. Therefore, addressing proper hand-hygiene for food growers, processors, and handlers can avoid many illnesses. The proposed system can bring awareness to employees whose job requires consistent hand-hygiene, regardless of their age.

One key difference between the system we develop here and the methods in [5,6,13] is that our cameras are not overhead, and the sink area where handwashing occurs may have extraneous objects. This increases the image and video processing challenges, but also opens up opportunities for the system to observe other food handling activities. Another key difference is that we incorporate advances from deep-learning, action recognition, and action detection that have all been developed since the previous systems.

### 2.2. Background in Video Analytics and Computer Vision

**Action recognition:** Action recognition is the task of identifying the action class inside a video that contains only a single action. Initial explorations applied a detector to locate salient areas [18] and computed descriptors to extract discriminative information about the actions [19]. These so-called hand-crafted features, including the Improved Dense Trajectory (IDT) [20] were soon replaced by deep learning methods, since with the support of sufficient training data deep learning methods have significantly better performance. Convolutional neural network (CNN) models, including AlexNet [21], VGGNet [22], and ResNet [23], perform well for an image classification task, and can also be used as a spatial feature extractor for video processing. These features can then be input to a temporal model such as an LSTM [24], BLSTM [25], ConvLSTM [26], or TRN [27], to generate the final prediction result. The use of 3D CNNs, which process video as fixed size input volumes has also been applied to action recognition, including C3D [28], I3D [29], T3D [30], and P3D [31]. However, due to their structure, these systems do not lend themselves toward flexible designs; they require an input with fixed temporal length and have many parameters to train.

**Action detection:** In practice, a video is highly unlikely to contain a single action, and the start and end time of an action within a video is typically unknown. Action detection addresses this problem by processing untrimmed videos with the goal not only to recognize all actions, but also to localize each action temporally. One typical approach is inspired [32] from object detection to use temporal proposals for action detection. Within this basic framework, several approaches have been proposed: the temporal structure of each action instance is modeled using a structured temporal pyramid in [32]; a Temporal Context Network temporally localizes and ranks proposals in [33]; a Temporal Action Localization Network is introduced in [34], while [35] identifies relevant regions using temporal boundary regression for actions. Another approach applies temporal convolution with either one stage [36] or multiple stages [37] to build an encoder–decoder structure to solve this problem. For our hand-hygiene processing, we focus on actions that happens inside a short duration; for example, rubbing the hands with water is only necessary for a few seconds. Thus, we choose to densely process the entire untrimmed video with an action recognition model to achieve hand-hygiene action detection.

**Domain adaptation:** Under ideal situations, the action recognition model assumes the training and test data come from the same data distribution. In reality, it is impossible to collect data from all potential scenarios in which the model might be deployed. Therefore, a performance drop is unavoidable if the CNN model is constructed on one scenario and deployed for another. The research field of domain adaption focuses on solving this issue, where the goal is to construct an initial model using data from a *source domain*, and later adapt the model to a new *target domain,* [38]. Unsupervised Domain Adaptation (UDA) further assumes that no labels are available from the target domain. UDA is useful for realistic applications with limited availability to the target domain prior to deployment, and has been applied to image classification [39,40] and video classification [41,42].

### 2.3. Implications for Designing a Cross-Scenario Hand-Hygiene System

For our hand-hygiene application in an open-room environment, UDA is critical. While it is reasonable to record and annotate data from one or two scenarios during the design of the system, it is prohibitive to label data for any new environment prior to deploying the system. We are particularly interested in UDA for *cross views* or *cross scenarios* [43,44].

We focus in this paper on three main strategies for UDA in our cross-scenario hand-hygiene application: defining a common action set across all scenarios of interest, selecting a spatial region of interest (ROI) to process instead of processing the entire image, and building a robust feature representation. For the first strategy, in addition to limiting the action set to the hand-hygiene actions that are common in all the scenarios we consider, we also take the approach to build a classifier to reject non-hygiene actions prior to analyzing the hand-hygiene actions. More detail on this strategy appears in Section 4.3.2.

For the second strategy, we consider confining a CNN model to operate only on a region of interest (ROI). This approach is inspired by explorations into Human–Object Interaction (HOI) [45] that demonstrate that focusing on the spatial region of an image where a person interacts with an object can be highly effective. The task of HOI is to locate both the human and an object and predict an interaction class category. A baseline framework of HOI consists of object, human, and interaction streams [46]. For object and human streams, a *hard attention* is created by cropping an ROI at the locations of both the object and human to force a CNN model to consider only information on these regions. Section 4 describes the regions we consider in more detail, and Section 7.1 and Section 7.2 provide information about how these are incorporated into our system design.

The above two strategies are used in both our baseline system and in our hierarchical system with multiple input modalities. The third strategy, to build robust feature representations, is a focus for the hierarchical system. Considering hand-to-object interactions provides one method to create a robust feature representation; another is to consider multiple input modalities. While it is most common to use the RGB input modality to process images or videos, other modalities have also been considered for action recognition. Optical flow, which captures pixel-level motion information, has been combined with RGB for action recognition, including in two-stream networks [47] and temporal segment networks [48]. Skeleton joints, which can capture crucial semantic body joints, have been shown to provide rich information for action recognition, using a spatial temporal graph convolutional network [49] and an end-to-end attention model [50]. They have also been used to indicate important discriminative regions in the image [51], when combining both skeleton joints and RGB modalities. Modalities such as optical flow and human skeleton joints are capable of maintaining motion or human-only information, which effectively removes any distractions of irrelevant objects and creates robustness against different scenarios. Conveniently, large-scale human action datasets like UFC101 [52] and NTU RGB+D [53] provide extensive training data for deep learning models. This allows us to generate human skeletal/joint information for our hand-hygiene videos by directly applying a pre-trained OpenPose [54] network. Thus, not only can we take advantage of the multiple modalities to compensate for potential weaknesses in the RGB modality, these approaches also provide a feature representation that is more robust to appearance variations, even without having data available from the target environment.

## 3. Method

In this paper, we apply an engineering design process. There is no one best approach to engineering design, although most include the following: Ask, (research), imagine, plan, create, test, improve. Moreover, it is iterative and one can revert back to a previous stage at any point. In the course of developing our final hierarchical multiple-modality system, we conducted these steps:

Step 1:   Define the problem and gather appropriate data;
Step 2:   Label the data, which helps assess the appropriateness of the visual task;
Step 3:   Design and implement a baseline system;
Step 4:   Evaluate the baseline system;
Step 5:   Redesign both to improve the robustness of our feature representations and to leverage advances in computer vision;
Step 6:   Evaluate the improved system.

We discovered during Step 4 that our baseline system performed poorly when it was trained using data from one scenario but applied to data from another scenario. Therefore, Step 4 was actually broken into smaller steps, namely:

Step 4(a): Apply Grad-CAM to better understand the weak design;
Step 4:(b): Apply a hidden-patch experiment to verify that the baseline system learns to pay attention to extraneous information.

The redesign, Step 5, was informed by the results of these experiments, and resulted in a hierarchical system that both improves the robustness of our feature representations and to leverage advances in computer vision. Therefore, Step 6 is also decomposed into smaller steps:

Step 6(a): Evaluate each subsystem of the hierarchical system separately;
Step 6(b): Evaluate the overall hierarchical system.

## 4. Dataset: Class-23 Hand Hygiene Dataset

In this section, we introduce our new **Class-23** hand-hygiene dataset. We describe the video collection steps and data pre-processing. Then, we discus the definitions of the hand-hygiene tasks for this dataset. Finally, we describe the ground truth labeling and data availability for action recognition and detection tasks.

### 4.1. Rationale for a New Dataset

As we described in our previous work [3], the published available food-related datasets have a different focus than our food safety research. The Georgia Tech Egocentric Activity (GTEA) [55] and Activities of Daily Living (ADL) dataset [56] collected a dozen of people’s daily activities in a home scenario. The MPII cooking activity dataset [57], 50salads dataset [58], and Breakfast Actions Dataset [59] recorded food handling and cooking in a home scenario. However, professional food safety facilities have a different configuration than the general home kitchen. To simulate the food safety environment, in our previous work we collected 100 participants who performed hand-hygiene in college bathrooms [3,7]. In this paper, we extend our data collection scenario into a professional food handling laboratory.

Our dataset provides video data that can support both action recognition and detection tasks. For action recognition, each of these hand-hygiene or non-hygiene actions are considered to be trimmed video clips. For action detection, the untrimmed videos include all actions of a person’s hand-hygiene steps from start to end.

### 4.2. Data Collection

In our new data collection, we invited 23 students to participate; these students were enrolled in a cooking class at Purdue University, hence the dataset name **Class-23**. The participants were all undergraduate students at Purdue University, with a likely age range from 18 to 22 years old. Six were male and 17 were female. All participants were healthy, and none had a handicap that prevented them from following instructions.

In each class session, students were required to perform hand-hygiene before starting the lab portion of the class. To reduce waiting time, students were split into two groups to perform hand-hygiene in two different rooms; we called these Room1 and Room2. Each room has one sink, and students lined up to wash hands one after another. All students followed the strict laboratory policy of wearing a white lab coat and a bouffant cap during the entire lab section. Students were aware of standard hand-hygiene procedures defined by the World Health Organization [2], although they may not have followed them precisely. During the video recording, students performed hand-hygiene without supervision from the instructor. Therefore, *non-hygiene* behaviors such as talking to each other and walking around the room are also captured in our data collection.

**Figure 1:**
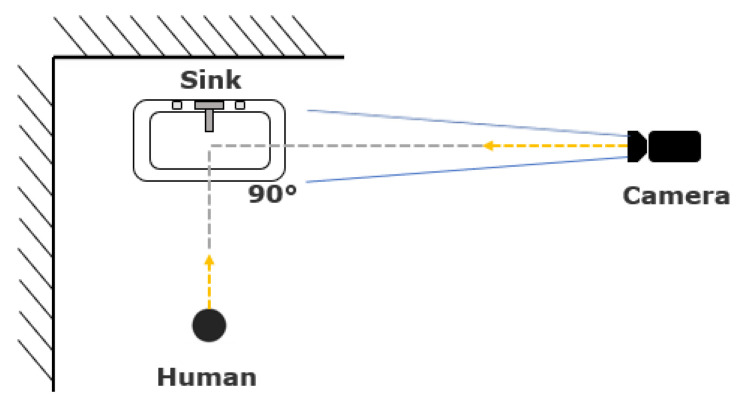
Room1 layout; Side-camera view with a 90-degree angle.

**Figure 2:**
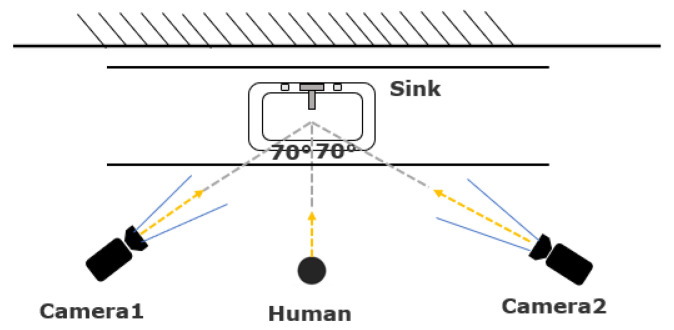
Room2 layout; two side-camera views with an approximately 70-degree angle.

The two rooms have distinct layouts; the location of the sink and the configuration of objects around the sink are different, which affected our camera placement. Figure 1 indicates the layout of Room1, where the sink is located at the corner of laboratory. Thus, we could only place the camera on one side of the sink for video recording. The angle between the lines connecting the sink and camera, and connecting the camera and the person was approximately 90 degrees. However, as indicated in Figure 2, the sink in Room2 is inset into a countertop, which prevented us from placing the camera at 90 degrees. Instead, the two cameras were placed on either side of the person (labeled “human” in the figure), with a viewing angle of about 70 degrees for each camera.

**Figure 3:**
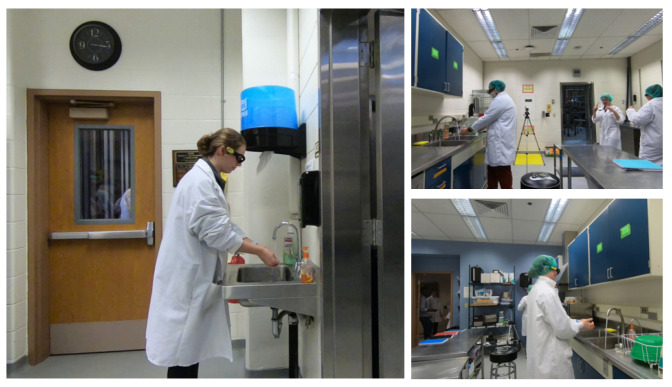
Data image; **Left:** Room1 Camera1. **Right top**: Room2 Camera1. **Right bottom:** Room2 Camera2.

Each of the three cameras were GoPro cameras mounted on a tripod. Each video has 1080p resolution at 30 fps. We collected 5 days of video data, each during a session of the class, for our **Class-23** dataset. (Data collection stopped when the class shifted unexpectedly to all-remote learning in March 2020.) Based on the way the group was split between the two rooms, the number of students in each room was not consistent from day to day. A frame from each camera is shown in Figure 3. Due to the clear variability across these three camera views, we consider the captured videos as corresponding to three distinct scenarios: Room1 Camera1 (R1C1), Room2 Camera1 (R2C1), and Room2 Camera2 (R2C2).

### 4.3. Data Processing and Labeling

#### 4.3.1. Creation of Untrimmed Videos

The recorded videos were pre-processed to remove those time periods where students or staff unintentionally occlude the camera view for an extended period. We also removed videos in which the tripod was incorrectly positioned. All cameras started recording before students arrived and were manually stopped after the last student finished hand-hygiene. To remove unnecessary content, we segment each video by person. Each so-called untrimmed video starts when a person begins hand hygiene and ends after that person finishes. There are a total of 105 untrimmed hand-hygiene videos across the three cameras; 30 are in R1C1, 28 in R2C1, and 47 in R2C2. Each untrimmed video includes all the steps of one person’s hand hygiene on that day.

#### 4.3.2. Defining the Set of Actions to Be Labeled

Defining the set of actions to be identified is a critical step after data collection that governs the performance of the subsequent classifiers. The set of labels must be reasonably complete before the annotation for the first experiment starts, or the annotator must go back and re-annotate, which is an unpleasant task and wastes a lot of time.

For video analytics of hand-hygiene, we have defined [3] three sets of tasks based on their level of difficulty: detail level, standard level, and detection level. Detail-level hand-hygiene recognition requires recognizing 12 different hand-hygiene actions, some of which involve subtle hand and finger motions. These are hard to observe even with an egocentric camera, so this detail-level hand-hygiene recognition is not a feasible task for the **Class-23** dataset which uses a side-view third-person camera. Therefore, our goal here is to focus on the standard-level hand-hygiene task, which includes 6 different hand-hygiene actions: *touch faucet with elbow*, *touch faucet with hand*, *rub hands with water*, *rub hands without water*, *apply soap*, and *dry hands with paper towel*.

However, due to the behavior of the students, the action sets present in the recorded videos are not the same. This requires us to adjust the action types to coincide with what was actually observed. Room2 Camera1 (R2C1) and Room2 Camera2 (R2C2), no students dry their hands with a paper towel, because the location of the paper towel is never within camera view, and there are only a few examples of this action in Room1 Camera1 (R1C1). Moreover, this action is best viewed using an egocentric camera [3] because the paper towel location is uncertain and the participant could walk while wiping their hands. Therefore, for this dataset, we will consider this action, *dry hands with paper towel*, to be a non-hygiene action. Moreover, it is quite surprising that none of the students in five days of recording ever performed the action of touching the faucet with their elbow. Therefore, we will only focus on four types of hand-hygiene actions in this work: *touch faucet with hand*, *rub hands with water*, *rub hands without water*, and *apply soap*.

#### 4.3.3. Labeling and Creation of the Trimmed Videos

In our **Class-23** dataset, each frame is labeled by a human expert to indicate the hand-hygiene actions. In addition, non-hygiene actions such as *swinging hands*, *grabbing a paper towel*, *drying hands with paper towel*, and *camera occlusion* are also labeled for each frame. These actions and the other non-hygiene actions are summarized in Table 1, which also indicates which of the hand-hygiene actions are considered to be hand-to-hand (H2H) actions and which are considered to be hand-to-object (H2O). Using these labels, trimmed videos are created that contain only one action per clip.

**Figure 4:**
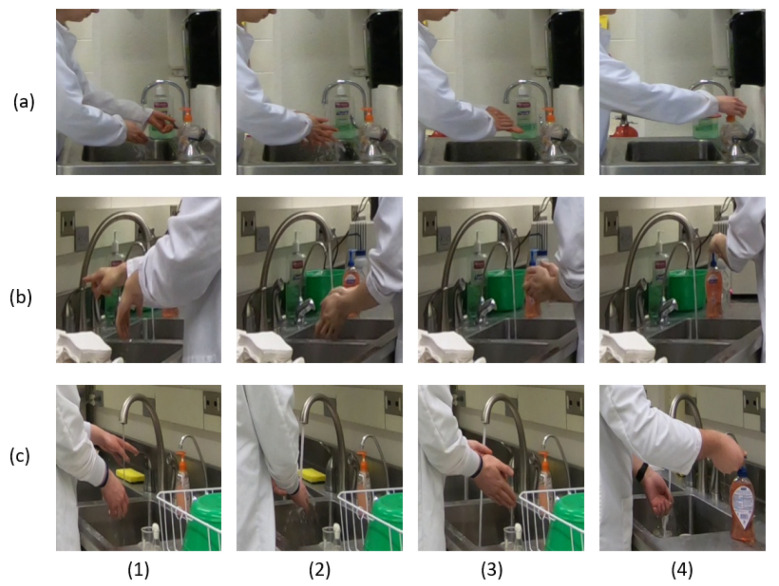
Cropped ROI of sink with four hand-hygiene actions. Columns: (**1**) touch faucet with hand, (**2**) rub hands with water, (**3**) rub hands without water, (**4**) apply soap. Rows: (**a**) Room1 Camera1, (**b**) Room2 Camera1, (**c**) Room2 Camera2.

To assist the system in focusing on the one student who is at the sink and performing hand hygiene, we manually label the sink region of interest (ROI). This labeling happens once per day per camera. We crop and resize this ROI to 224 × 224 pixels, and for the remainder of this paper unless specifically indicated otherwise, these cropped and resized ROI images or videos are the default input to all experiments. Examples of these cropped ROI images are shown in Figure 4.

**Figure 5:**
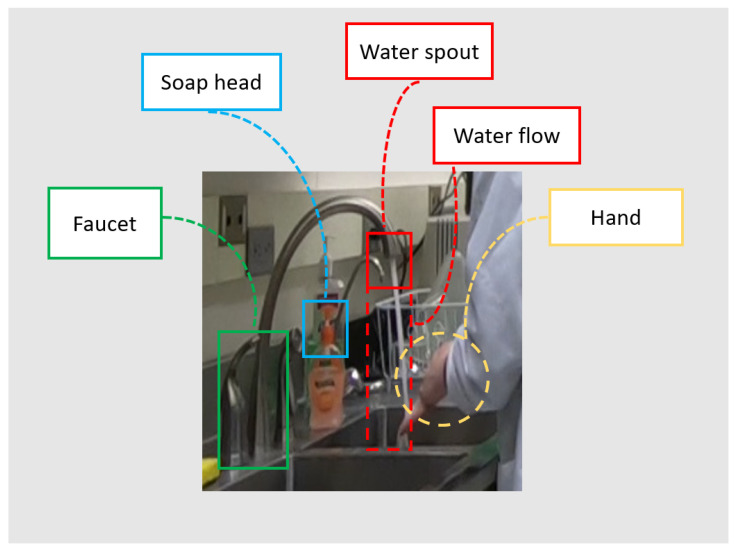
Regions of interest, **Solid red rectangle**: water spout. **Dotted red rectangle**: water flow. **Blue rectangle**: soap head. **Green rectangle**: faucet. **Yellow circle**: hand region.

We also label additional ROIs for the scenes, to focus the attention of the models to the spatial regions we know are important to hand hygiene. Table 2 lists the ROIs we consider here, and they are illustrated in Figure 5. Hand ROIs are generated using OpenPose [60], while all other ROIs are manually annotated. Note that all the ROIs, except for the hands, the sanitizer (whose ROI is not used in our final system), and the soap are stationary objects recorded by a stationary camera. Therefore, these ROIs only need to be labeled once when the camera is positioned, so this is a feasible approach for a cross-scenario system.

Ideally, these regions could be found using an object localization method such as Faster R-CNN [61], You Only Look Once (YOLO) [62], and SSD [63]. However, we chose manual annotation in this paper, which directs the focus of this paper to exploring the general feasibility and design of a system for cross-scenario hand-hygiene assessment across different rooms with different layouts, illumination, and object configurations.

### 4.4. Training, Validation and Testing Data Creation

The trimmed and untrimmed videos are each partitioned into training, validation, and testing sets. Trimmed videos are used for action recognition, and untrimmed videos are used for action detection. As is typical for machine learning, during training the model is evaluated on both the training data and on the validation data. Training continues while both results are improving, but when the performance on the validation data begins to decrease, training is stopped. This is one way to limit overfitting. The validation set, used only during algorithm development, can also be used to tune hyperparameters during training. The testing data are used exclusively to evaluate the model once training is complete.

After removing the untrimmed video clips with heavy occlusion or an incorrectly positioned tripod as discussed above, we had 62 training, 9 validation, and 34 testing videos. Each includes one person washing their hands from beginning to end. Trimmed video clips corresponding to these were categorized identically. For example, untrimmed training videos were processed to become trimmed training videos, with each trimmed video containing a single action. The resulting number of trimmed videos for each scenario are shown in Table 3. Untrimmed videos were not used for training or validation, only for testing the complete hierarchical system. There were 9, 10, and 15 videos used for testing purposes in Room1 Camera1, Room2 Camera1, and Room2 Camera2, respectively.

## 5. A Baseline System for Hand-Hygiene Action Recognition Using RGB Inputs

Here we present a baseline system for using a CNN with RGB inputs to recognize hand hygiene actions in trimmed videos. The goal here is to explore the degree to which this simple model can adequately recognize hand hygiene actions across the three scenarios. In addition, this exploration provides useful insights into how well this generic action-recognition system can detect the actions in our application. If performance is unacceptable even in the same-scenario case, then an effective solution to the application may not be feasible. If the performance is acceptable in both the same-scenario and the cross-scenario case, then further explorations are unnecessary.

We present the baseline system and some minor variants in Section 5.1 and describe our experimental method and results in Section 5.2. The experiments consider the case where the training data come from the same scenario (room and camera) as the testing data, as well as the case where the training data come from a different scenario than the testing data, a case we term the cross-scenario case. For the same-scenario case, we explore both a spatial-only and a spatial+temporal model, and also consider a classifier for two different sets of actions, one without non-hygiene actions and one with non-hygiene actions.

### 5.1. Baseline Hand-Hygiene System Description

In this subsection, we present a baseline CNN-based system for hand-hygiene action recognition, and several minor variants on the baseline system. The baseline system uses a spatial-RGB input to CNN to decide among the four hand-hygiene actions that are present in all three of our scenarios (see Table 1). This system is termed “ResNet50 (H)” here, where the H indicates that we consider only hand-hygiene actions. This baseline model is simply a 2D CNN ResNet50 model that has as input a single frame of a video. A decision is made for each frame in the video, and a final decision for the trimmed video uses majority vote within the video to identify an action.

The first two variations incorporate temporal modeling into the baseline system. We consider two temporal modeling methods: combining a CNN with Long Short-Term Memory (LSTM) [24] or with a Temporal Relational Network (TRN) [27]. These systems are termed “ResNet50+LSTM (H)” and “ResNet50+TRN (H)”. Temporal models similar to these have been shown to improve the performance of 2D CNNs in other applications [64,65]. Temporal modeling requires selecting a fixed number of frames, *k*. A variety of methods to select these frames are common. For example, the Temporal Segment Network (TSN) [48] cuts a video input into segments and applies random sampling within each segment to obtain a fixed length input. Other methods include uniform sampling or selecting consecutive frames at the beginning of a video. For hand-hygiene action recognition, we use k=10 consecutive frames. This is short enough to enable a real-time implementation in the future, and long enough to capture posture, appearance, motion, and motion consistency for these actions [3]. The 2D CNN ResNet50 is used as a feature extractor by removing the last fully-connected layer; each temporal model accepts these features as input. The system uses a sliding window of *k* frames, forms a prediction for every frame, and uses a majority vote within the video to identify the action.

The final minor variation is to use a similar spatial-only system as the baseline, but to decide among five actions, where a generic non-hygiene action is added to the set of four hand-hygiene actions. During daily hand-hygiene, it is unavoidable for a participant to perform a variety of unexpected actions other than hand-hygiene. These actions are unlikely to contribute one way or another to the quality of the hand hygiene. Therefore, it is useful to understand if these non-hygiene actions affect the performance of the baseline system. This system is otherwise identical to the “ResNet50 (H)” system and is termed “ResNet50 (H+N)”, where the +N indicates it also considers non-hygiene actions.

The baseline system and the variations are trained using a similar strategy. All ResNet50 models are initialized with the pre-trained weights from ImageNet [21], and weights are fine-tuned using our data. For the two spatial-only models we randomly select frames from the training set videos. To train the spatial-plus-temporal models, we randomly select a start frame and consecutively sample *k* frames to create an input for the temporal model.

The CNN ResNet50 model is trained with 350 epochs with batch size 32 and an initial learning rate 0.001, which decreased by a factor of 10 at 200 and 300 epochs, using a Stochastic Gradient Decent (SGD) optimizer. For the loss function, we performed an initial experiment to compare the binary cross-entropy loss and the cross-entropy loss. We chose cross-entropy loss because it obtained better performance on the validation set. To avoid over-fitting, data augmentation using a multi-scale crop and random horizontal flip was applied during the training stage as described in [66]. During training, each model is applied to training and validation data. When performance on the validation data worsens, we stop training to prevent over-fitting. See Section 4.4 for details on training and validation data.

### 5.2. Performance of Baseline System and Its Variants

In this section, we take an experimental approach to address the following questions:

- Does the baseline system perform well in the same scenario case?
- Does adding temporal information to the baseline system improve performance?
- Is performance of the baseline system degraded if it must also decide about non-hygiene actions?
- Does the baseline system still perform well in the cross-scenario case?

We begin with the same scenario situation; models are tested using data from the same environment as they were trained on. The testing data are unique from the training data as described in Section 4.4. Table 4 shows the performance of the baseline action-recognition system and all its minor variants in the same-scenario case for the four hand-hygiene actions of interest. As can be seen, all systems perform similarly, and all achieve over 90% accuracy in each environment. Temporal models have little effect on performance, and adding non-hygiene actions also has little effect on the ability to recognize the hand-hygiene actions. From the results in this table, we conclude that to achieve good performance in the same-scenario environment, even the simple baseline spatial-only model is effective. This indicates that the overall problem of hand-hygiene action recognition with these four actions lends itself to a simple solution, provided the deployment environment is similar to the training and development environment.

Next, we explore the ability of the baseline system to generalize to unseen environments. Specifically, we consider the case where the training data come from a different scenario than the testing data. We term this the cross-scenario case. We apply same baseline models as above, each trained in one environment, on the testing data from the other environments. All testing sets are identical to those used in Table 4 for a direct comparison. Table 5 presents the cross-scenario testing results. Note that the accuracies of the model trained and tested in the same scenario (shown in bold) are identical to those in Table 4. However, there is a dramatic and unacceptable performance drop when a model trained in one scenario is applied in another scenario. Incorporating a temporal model does little to improve the situation, although the TRN model outperforms the LSTM model almost always.

### 5.3. Discussion about Baseline System

The experiments above address the question: does a straightforward spatial-only baseline system solve the video recognition task? The results clearly answer this question, that yes, it is sufficient in the same-scenario case, but also no, it is completely inadequate when applied in the cross-scenario situation. Unfortunately, this loss in performance is not surprising. It is well known that data collected for a particular task inevitably describe only part of the task; this is termed the dataset bias problem [67]. Capture bias depends on factors such as camera view, illumination conditions, and background scenes, all three of which are present in the scenarios we recorded for our **Class-23** hand-hygiene dataset. While dataset bias is certainly a major contributor to the performance drop in the cross-scenario case, further explorations can provide insights into more specific causes and hence into potential solutions.

## 6. Exploring Reasons for Poor Performance of the Baseline System

In this section, we describe two experiments to explore the causes of the poor performance of the baseline system in the cross-scenario case. The first uses Grad-CAM [68] to highlight inconsistencies between what the model is focusing on and what a human watching the scene would focus on to make a decision. The second experiment uses a hidden-patch approach to verify the hypotheses formed from the first experiment. We include these in this paper because understanding model failures is an important component of designing an effective, practical system. Because the hidden-patch experiment is designed based on observations from the Grad-CAM experiment, we describe their methods and results sequentially.

### 6.1. Grad-CAM Exploratory Experiment: Method, Results, and Discussion

In the first experiment, we use Grad-CAM [68] to generate a saliency map that visualizes the discriminative area that our CNN model uses to make its prediction. Grad-CAM, or Gradient-weighted Class Activation Mapping, creates a visualization of which pixel regions in a particular image contribute most heavily when computing the classification label using a CNN. In our experiment, we apply Grad-CAM on the conv_5x layer of ResNet50 to generate a saliency map for several instances of the *rub hands with water* action.

**Figure 6:**
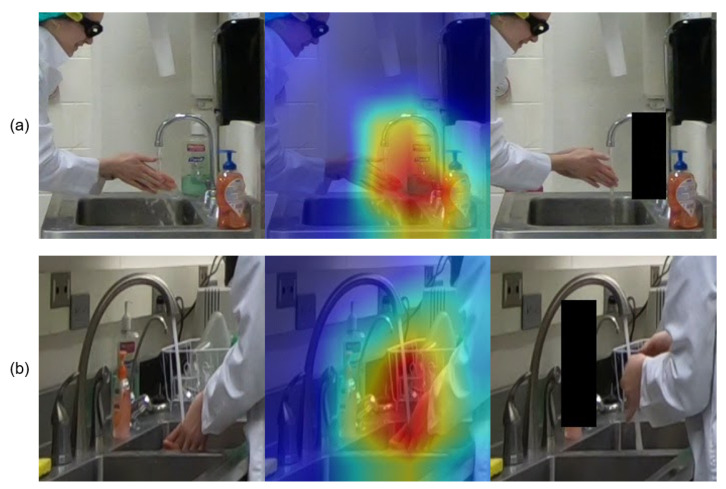
*Rub hands with water* action. Left column: images; middle column: saliency map. Right column: image with black patch hiding the hand sanitizer bottle. Row (**a**): Scenario Room1 Camera1; Row (**b**): Scenario Room2 Camera1.

The top and bottom rows of Figure 6 show two different scenarios depicting the *rub hands with water* action. Both images produce the correct prediction when using the 2D CNN ResNet50 corresponding to their scenario. We see from the middle column that the discriminative region for R1C1 not only covers *hands* and *waterflow*, but also include objects *sanitizer*, *water spout* and *faucet*, which are completely irrelevant objects for this action. In contrast, the discriminative region for R2C1 mostly covers only the *hands* and *waterflow* regions, which is more consistent with how a person would decide on the action.

Thus, it appears that in some scenarios, the discriminative region learned by the CNN during the weak supervision training process might involve extraneous objects. These extraneous objects become a visual cue that misleads the CNN when it is applied on data from other scenarios and limits the capability of the model to be relevant for cross-scenario recognition.

### 6.2. Hidden-Patch Exploratory Experiment: Method, Results, and Discussion

Our second experiment, using hidden patches, is constructed to confirm our observations below based on the results of the Grad-CAM experiment. Hiding patches of an image, either during training [69], testing [70], or both [71], has been shown to be a valuable tool. During training, hiding patches can force a CNN model to broaden its attention to additional discriminative regions. During testing, if hiding patches creates a distinct drop in performance, this may reflect the importance of that hidden area to the model.

For this experiment, we focus on verifying the impact of the hand-sanitizer bottle on the action *rub hands with water*. We cover that object with a black patch for all testing images from both R1C1 and R2C1, as illustrated in the rightmost column of Figure 6. We apply the 2D CNN ResNet50 models of each scenario on their own hidden-patch images.

To highlight the change in the CNN result, before and after hiding the patch, we consider the raw prediction score from the CNN before applying the softmax function. Table 6 shows the average prediction scores for both scenarios when testing only the videos labeled as *rub hands with water*. Note that since these values are prior to softmax, they could be any real number. Larger positive numbers indicate stronger confidence. Average confidence scores are reported for each of the four actions. As can be seen, the confidence score for *rub hands with water* in scenario R1C1 drops 2.32 with the hidden patch, while the confidence score for scenario R2C1 drops only 0.55. Interestingly, neither model changes their action predictions, because the hidden patches do not change which action has the highest confidence score when testing on the same-scenario data. However, this does verify that the model trained for the R1C1 scenario relies heavily on the irrelevant sanitizer bottle to decide the action, and this raises significant concerns about the ability of the RGB-only model to perform well in the cross-scenario action recognition for our application.

The above result highlights a fundamental weakness of weakly-supervised learning for action recognition. In weakly-supervised learning, each action video is only labeled with the action class label, and the model must somehow identify discriminative regions during the training process on its own; there is nothing to prevent the model from relying on an irrelevant object as a discriminative visual cue of an action. Since the environment around the hand-washing sink could contain many irrelevant objects and these could change or move over time, the RGB CNN model presented in this section is unlikely to generalize well across a wide range of scenarios. Thus, the next step is to consider other methods to build an effective action detection system for hand-hygiene in an open-room environment.

## 7. Hierarchical Action Detection System Using Multiple Modalities for Cross-Scenario Hand Hygiene

Informed by the inability of the baseline system to generalize and perform well in the cross-scenario situation, in this section we present a new hierarchical system. This system is designed to achieve two goals: improve the feature representations so they are robust across scenarios, and leverage advances in computer vision beyond a spatial-only CNN. First, we decompose the overall classification problem into a series of smaller, simpler classification problems. Second, we choose an input modality for each simpler classifier that is targeted toward the specific task.

### 7.1. The Overall Hierarchical System

**Figure 7:**
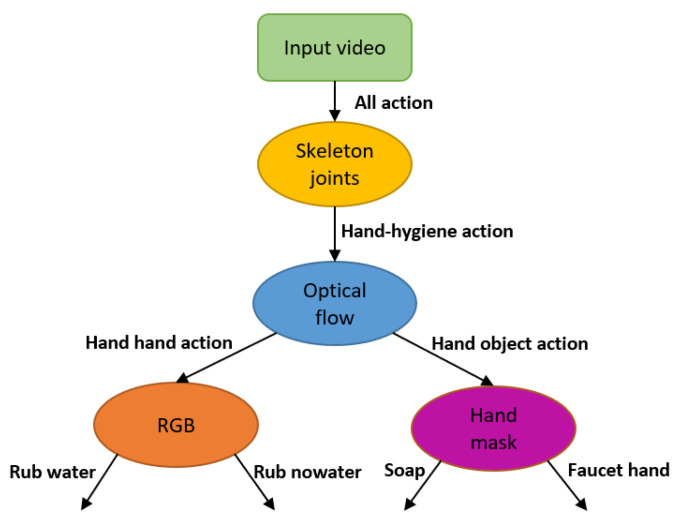
Hierarchical action recognition with multi-modalities.

**Figure 8:**
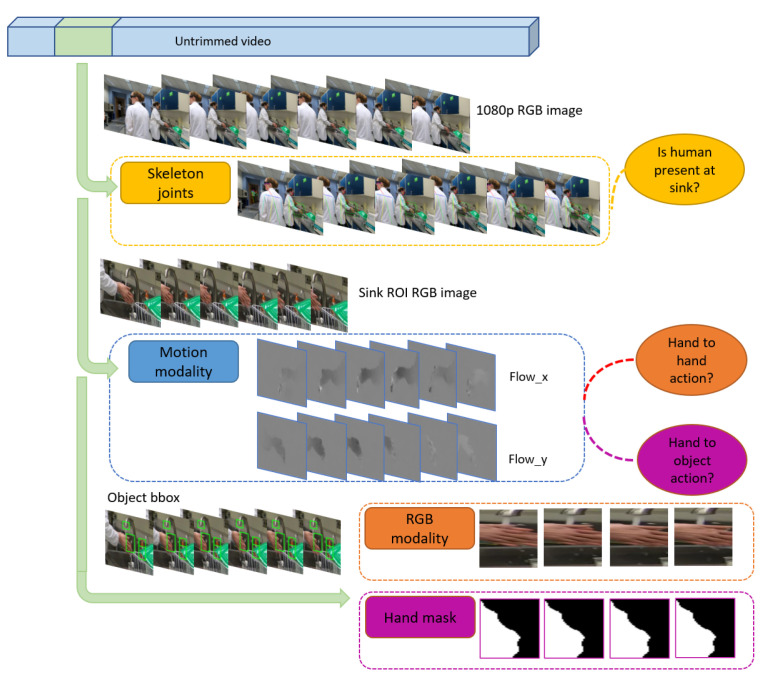
Cross scenario action detection.

Our hierarchical system is shown in Figure 7, with a more detailed system structure in Figure 8. This hierarchical approach enables each sub-classifier model to have its own feature space and architecture, and avoids a highly intertwined model. The system relies on having the sink ROI already labeled. Both the overall system and each subsystem can perform either action recognition or action detection. For both tasks, the input video chunk is a set of k=10 consecutive frames.

First, for a given chunk of video frames, either from an untrimmed video or a trimmed video clip, we first detect the skeleton joints present in the full 1080p image by applying an unsupervised skeleton-joint modality model to reject non-hygiene actions for which there are no upper-body joints appear in the sink ROI. Second, using the pixels in the sink ROI only, chunks of video frames which are not rejected are then processed by the model using the optical flow modality to distinguish between H2H and H2O actions. Third, chunks of video in these two categories are then processed by either an RGB model or a hand-mask model. The RGB model decides among H2H actions *Rub with water* and *Rub without water*, while the hand-mask model decides among H2O *Apply soap* and *Touch faucet with hand*. Note that the hand-mask model requires ROIs for the water spout, faucet, and soap head. The final outcome of the hierarchical system is a prediction of one of the four hand-hygiene actions.

For action recognition from a trimmed video, an action decision is made for each window of 10 frames, and the final system prediction result for a trimmed video is obtained by choosing the action that has the largest the average among all the softmax-ed scores for that video. For action detection applied to an untrimmed video, actions are decided for each second. The action for one second, which comprises 30 frames, is determined by the most frequent action among the 10-frame chunks for that second. This approach to action detection is more efficient for our hand-hygiene task than more typical approaches for action detection [32,37]. Those methods create temporal proposals or apply an end-to-end training structure. However, for us, the duration of the entire set of actions is typically only one or two minutes long, which is much shorter than the applications those methods address, where the untrimmed video might last half an hour or more.

The overall hierarchical system does not require training; however, three of the subsystems do. The required training is described below as each subsystem is introduced.

### 7.2. Subsystem Descriptions for Each Modality

In this section, we present each subsystem for our hierarchical system. Each subsystem relies on a single input modality and focuses on making one specific decision.

#### 7.2.1. Subsystem 1: Deciding among Hygiene and Non-Hygiene Actions Using Skeleton and Object Coordinates

In the first subsystem, we concentrate on two input modalities to decide among hygiene and non-hygiene actions. These are object coordinates and human skeleton joints. For object coordinates, we consider the location of three objects: *faucet*, *water spout*, and *soap head*. These are represented by the center coordinates of the corresponding bounding box. Although these could be identified using an object detector, as discussed in Section 4.3.2, here we apply manual annotation of these mostly stationary object locations. For skeleton joints, we are interested in the upper-body skeleton joints of a participant, namely *shoulder*, *elbow*, *wrist*, and *hands*.

**Figure 9:**
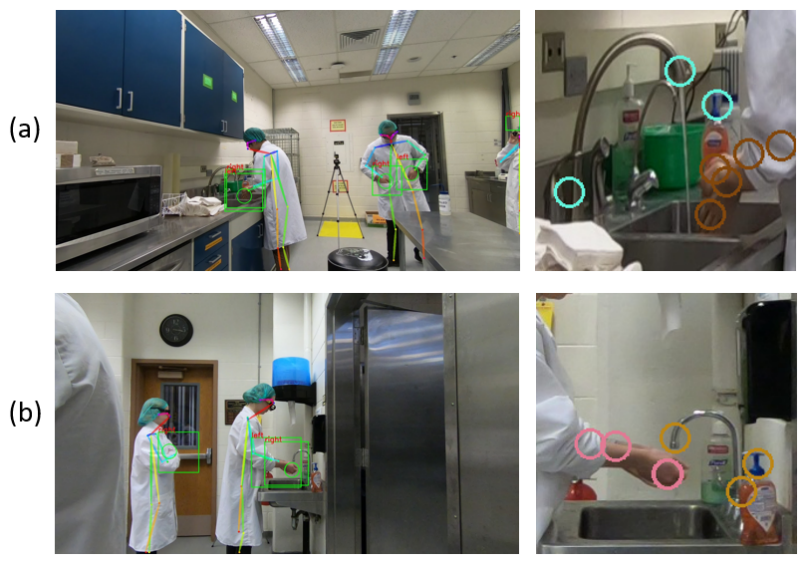
Coordinate detection results, left: OpenPose, right: object + upper-body coordinates; row (**a**) R2C1. Row (**b**) R1C1.

We apply skeleton joint detection using the complete 1080 image in which the entire body can be clearly viewed, because body detection performs well only if most of the body is visible. We use the OpenPose method [54] to detect 18 human body joints and 21 hand joints for each the people in the image, but retain only joints from the person around the sink. Among the 18 body joints, we only keep *shoulder*, *elbow*, *wrist*, and *hands* of both the left and right sides. The location of the 21 hand joints is averaged to obtain the coordinates of the hand. Two sample visualizations of coordinate detection are shown in Figure 9.

Using this subsystem, we classify actions as being non-hygiene actions or associated with hand hygiene. If hand or wrist joints are present in the sink ROI, then we conclude this is a hand-hygiene action; otherwise we decide it is an non-hygiene action. Note that this subsystem does not require any training using our data, as it relies on a pre-trained OpenPose method.

#### 7.2.2. Subsystem 2: Deciding among Hand-to-Hand and Hand-to-Object Actions Using Optical Flow Modality

In the second subsystem, we apply optical flow to decide whether a hygiene action is H2H or H2O (see Table 1). Optical flow, which describes motions for each pixel in the image, is a widely used modality in action recognition [47]. For our hand-hygiene actions recorded with a static third-person camera, optical flow can track the moving body parts of the participant, with a particular focus on the forearm, and can be used to reject information about static objects that might be scattered about the environment.

**Figure 10:**
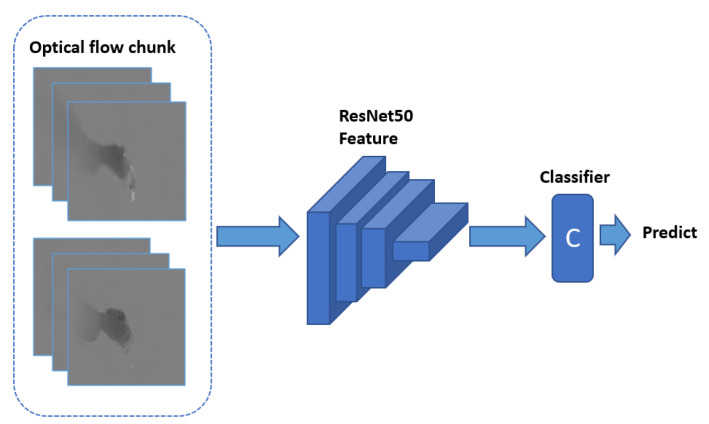
Model structure for the optical flow modality.

We compute optical flow using TV-L1 [72] and rescale the result to the range [0,255]. Similar to the temporal network structure described in [47,66], we select ResNet50 with weights pre-trained on ImageNet [73] as our model. The input is a stack of ten optical flow image pairs as shown in Figure 10. The first convolutional layer accepts 20 channels as input. The pretrained weight on that conv layer is averaged by its original 3 channels and repeated 20 times to match the parameter setting of the first convolutional layer.

The subsystem is trained to recognize if an action is H2H or H2O using data from each scenario; the models are referred to by the name of the associated scenario. Each CNN ResNet50 model is trained with 350 epochs with batch size 32 and an initial learning rate 0.001, which decreased by a factor of 10 at 200 and 300 epochs, using a Stochastic Gradient Decent (SGD) optimizer. We apply the cross-entropy loss function. To avoid over-fitting, data augmentation using multi-scale crop and random horizontal flip was applied during the training stage as described in [66].

#### 7.2.3. Subsystem 3: Deciding between Actions Apply Soap and Touch Faucet with Hand Using a Hand-Mask Modality

In the third subsystem, we apply hand masks to decide whether a H2O hygiene action is either *apply soap* or *touch faucet with hand*. Hand-hygiene actions contain body parts and often require interaction with objects that are in fixed locations, such as the faucet or sink. By applying image segmentation masks, we can discard redundant appearance information and maintain the shape and silhouette which is robust across scenarios. Therefore, we apply the mask information of human hands to recognize H2O actions: *touch faucet with hand* and *apply soap*. Since these two actions occur in the ROI of the two *faucet* and *soap head* objects, we crop the hand masks to contain only the ROI of these objects instead of using the whole image.

**Figure 11:**
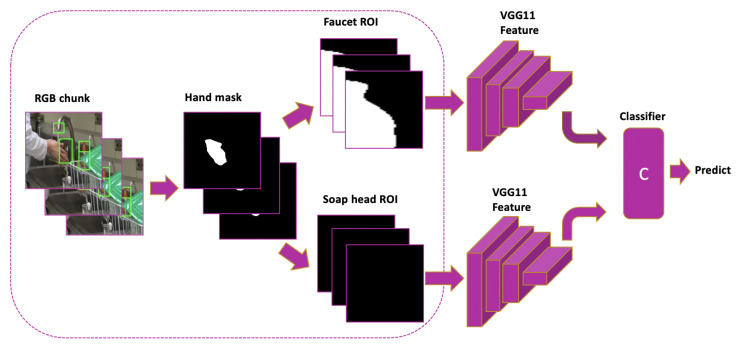
Hand mask modality model structure.

We generate human hand masks using the method in [74], which applies RefineNet [75] pretrained on the EYTH (EgoYouTubeHands) dataset for hand segmentation. Each hand mask image is a binary image. We resize the hand-mask ROI images to a fixed size of 224 × 224 and stack 10 frames to incorporate temporal information. As shown in Figure 11, each stack of hand-mask ROI images is input to a VGG11 [22] backbone with unshared weights; features are concatenated before the last fully-connected layer makes the final prediction.

The subsystem is trained to recognize if a H2O action is *touch faucet with hand* and *apply soap* using data from each scenario; the models are referred to by the name of the associated scenario. Each model is trained with 350 epochs with an initial learning rate 0.0001, which is decreased by a factor of 10 at 200 and 300 epochs.

#### 7.2.4. Subsystem 4: Deciding between Actions Rub Hands with Water and Rub Hands without Water Using the RGB Modality

In our final subsystem, we use the RGB modality to decide among the H2H actions *rub hands with water* and *rub hands without water*. Although the RGB modality was shown to perform poorly for distinguishing among all actions in the cross-scenario case in Section 5.2, the RGB modality has unique appearance information which is not captured by the other modalities. In particular, the appearance of the *waterflow* is crucial to distinguish between these actions.

**Figure 12:**
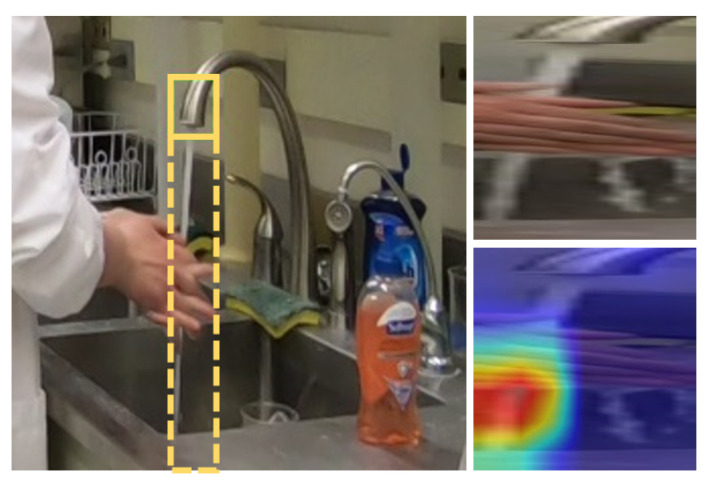
Hands overlap waterflow; **left**: water spout ROI extends to acquire waterflow. **right top**: resized waterflow ROI. **Right bottom**: waterflow ROI attention using Grad-CAM.

This subsystem applies “hard attention” by cropping a region of interest (ROI) of the waterflow to constrain the input RGB signal. The waterflow ROI is obtained by extending the human-labeled bounding box of the water spout down to the bottom of the image. This ROI is then resized into a 224 × 224 image. The process of creating the waterflow ROI is illustrated in Figure 12 (left and right top).

Our waterflow-based model applies a ResNet50 CNN using as input the stack of 10 RGB images associated with the waterflow ROI. The subsystem is trained to recognize if an H2H action is *rub hands with water* or *rub hands without water* using data from each scenario; the models are referred to by the name of the associated scenario. Each model is trained with 350 epochs with an initial learning rate 0.0001, and decreased by a factor of 10 at 200 and 300 epochs.

With the waterflow ROI applied, there are actually three situations of interest when comparing the two H2H actions. In the first case, the hands cut the waterflow and deflect the path of the water; in the second, the hands are not adjacent to the waterflow; and in the third, the hands occlude the waterflow but are not actually in the water. The first case corresponds to the action *rub hands with water*, and the second corresponds to the action *rub hands without water*. However, to learn to effectively distinguish these two actions, it is also crucial to be able to correctly process the third case. Unfortunately, in our data collection R1C1 and R2C1 do not include any examples of this third situation. For R2C2 though, the presence of situations where the hand occludes the flow of water forces the CNN model to recognize that the bottom area of water is a discriminative region. This increases feature robustness and assists in distinguishing these actions. This observation is supported by Figure 12 (Right bottom) which illustrates using Grad-CAM how that a model trained on R2C2 has learned to recognize that the bottom area of water is a discriminative region.

To improve this situation for R1C1 and R2C1, we use offline data augmentation to create additional training samples. We manually select several consecutive frames in those two scenarios where the hands occlude but do not intersect with the waterflow. These frames are repeated to create a 60-frame video clip for the situation *hands overlap with waterflow*. To be consistent with the existing amount of data in these two rooms for the two H2H actions, we created eight and three *hands overlap with waterflow* video samples to add into the R1C2 and R2C1 training sets, respectively.

Next, we apply adversarial learning to improve the domain adaptation [40]. Recall that using computer vision terminology, we can consider each of our three scenarios to be a *domain*, and our goal to achieve an effective model that performs well across scenarios can be considered as building a model using *source domain* data and labels that performs well in a *target domain*.

**Figure 13:**
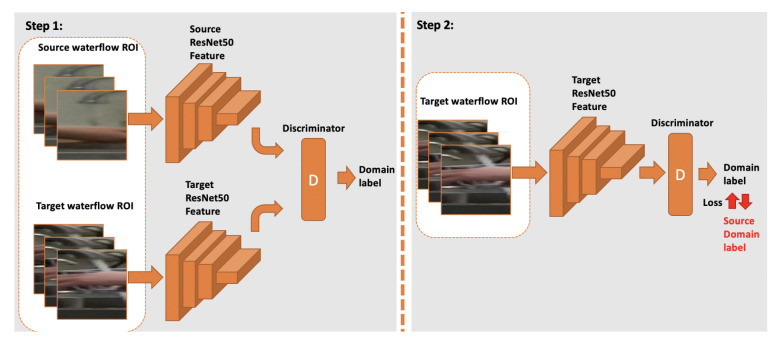
Target domain model training procedures.

For our adversarial learning, we use a discriminator in the training stage to assist in mapping the features from the target domain model feature onto source domain. We begin with a source-domain CNN model that has been trained using the labeled data from the source domain, as described above in this subsection. Then we follow two steps to alternately train a discriminator and a target-domain CNN, as illustrated in Figure 13. The goal of discriminator is to recognize which of the two domains (source or target) is associated with the input feature vector. The goal of the target-domain CNN is to produce a feature vector using target-domain data that fools the discriminator into thinking the feature vector came from the source domain. Step one starts by training the discriminator using the features from both domains, which are the result of two source- and target-domain CNN models. In step two, the target-domain CNN is trained to create output feature vectors that are more similar to the source-domain features.

The goal of this domain adaptation is to improve recognition between the two actions of rubbing the hands with or without water. However, effective training requires us to select only videos that contain these actions of interest and not the other actions. Unfortunately, to apply this strategy in operation, the target domain will not have been annotated. To address this problem, we apply the optical flow model trained in Section 7.2.2 to select those videos that contain these H2H actions and exclude those videos that do not. With this step, the domain adaptation procedure can focus on the actions of interest, which improves its effectiveness.

To implement domain-adaptation with adversarial learning, we choose ResNet50 with input of stack 10 frames as a CNN model for both source and target domain. The discriminator is a multilayer perceptron (MLP) with three layers. Both the target domain CNN and discriminator are trained with cross-entropy loss in combination with Adam optimizer using 0.00001 and 0.001 learning rates, respectively, for 350 epochs.

#### 7.2.5. Summary of Observations Regarding the Multiple Modalities

To summarize, each modality has its unique strengths. The skeleton modality, when computed from the original uncropped images and in combination with the location of the sink ROI, can be used to determine if there is actually someone standing at the sink, and hence to reject non-hygiene actions. The optical flow modality is effective at discriminating between H2H and H2O actions. The hand-mask modality, when combined with ROIs for the soap and faucet, can decide between the two H2O actions. Finally, the RGB modality, when supplemented with data augmentation and domain adaptation, can effectively discriminate between the two H2H actions. Note that our model for the hand-mask modality relies not only on our own data, but also on the data used to train the hand sementation algorithm [74]; similarly, the skeleton modality relies both on our data and on the data used to train OpenPose [54].

## 8. Cross-Scenario Results

We begin by describing performance results for action recognition for each subsystem individually in Section 8.1. Then, in Section 8.2, we describe the performance of the overall system for action detection.

### 8.1. Experiments and Results: Individual Subsystems

Here, we present the capability of individual modalities on *recognizing* hand-hygiene actions in the cross-scenarios case. Recall that a model been trained using data from each scenario. Each model is tested on the trimmed video from all three scenarios. The model is applied on every sliding window in the testing video. Each window has a fixed length of 10 frames and the step size between windows is 1 frame. The final prediction result for each subsystem is obtained by choosing the action that has the largest average among all the softmax-ed scores for that video.

#### 8.1.1. Subsystem 1: Experimental Method and Results

To begin exploring the effectiveness of detecting human joints in our scenarios, we apply OpenPose to the complete uncropped 1080p image and compute detectability results on the joints of the left and right sides of the person at the sink. As can be seen from Table 7, joints on the left side are well detected in R1C1 and R2C1, but the left elbow and hand are often missed in R2C2. Similarly, the right side is well detected in R1C1 and R2C2, but the right elbow and hand are often missed in R2C1. This is due to the camera angle which views the person slightly from behind, as shown in Figure 2. The detection rate for at least one hand or wrist is over 94%. The presence of missing joints motivated us to avoid the skeletal modality except to decide between hand-hygiene and non-hygiene actions. When we apply OpenPose to make this decision, as described in Section 7.2.1, the accuracy is 94.0%, 95.0%, and 96.4% for scenarios, R1C1, R2C1, and R2C2, respectively.

#### 8.1.2. Subsystem 2: Experimental Method and Results

The testing results of using optical flow for subsystem 2 are shown in Table 8. We observe that each model performs well when tested cross-scenario, with over 94% accuracy. Thus, we conclude that an optical flow model is effective across scenarios at deciding between H2H actions and H2O actions.

#### 8.1.3. Subsystem 3: Experimental Method and Results

The performance of applying the cropped ROI hand mask on cross-scenario recognition for subsystem 3 is shown in Table 9. The average detection accuracy of each action is computed from cross-scenario situations only, where a model is tested on all other scenarios except the scenario it was trained on. This approach provides good performance for recognizing both actions, especially using the model trained on R1C1. However, for scenario R2C1 it is difficult due to the camera angle and a lack of depth information to distinguish when hands are occluding an object but not touching it; this explains the reduced performance of the model trained for this scenario.

#### 8.1.4. Subsystem 4: Experimental Method and Results

To explore the performance for the RGB modality in subsystem 4; we consider using the waterfall ROI (indicated by “W”), data augmentation (indicated by “D”), adversarial learning (indicated by “A”). In addition, we also consider the so-called ideal case for adversarial learning, where we can use the ground truth of whether an action is a H2H action or not. We denote this final situation by “A(I)”. Results for these four cases are shown in Table 10.

Simply applying the waterflow ROI provides reasonable performance, particularly in the same scenario. When offline data augmentation is added, we obtain improved recognition accuracy for R1C1 and R2C1 models on R2C2 data. (Note there is no row for R2C2 W+D because data augmentation was not necessary for this scenario.) Adversarial learning further improves the performance in most of the cross-scenario cases, especially the for model trained on R2C1. Finally, when we consider the ideal case, where adversarial learning is based on ground-truth which would be unavailable in practice, we obtain excellent performance for most scenarios.

### 8.2. Results: Hierarchical System

In this section, we present the overall performance of our complete hierarchical system.

#### 8.2.1. Experimental Methods for Hierarchical System

For a comparative baseline to our hierarchical system, we choose the 2D CNN Resnet50 model with RGB inputs which has been described earlier in Section 5.1. In addition, to illustrate the performance of the overall method, we also present results for the ideal situation introduced in Section 7.2.4. Recall that ideal model used manual annotation of the target domain data to distinguish between H2H and H2O actions for the domain adaptation process.

We apply three evaluation metrics: frame-wise accuracy (F-acc), window-wise accuracy (W-acc), and task-wise accuracy (T-acc). Frame-wise accuracy directly compares the frame-level prediction result with the ground-truth label. To compute window-wise accuracy, we split the untrimmed video into non-overlapping fixed-length windows and compute the prediction result for the window as the average predication across all the frames in the window. The window-wise accuracy is then computed by comparing the window-level prediction with the ground-truth label for the window. This metric allows us to evaluate the effectiveness of our estimates of how long the H2H action *rub hands without water* lasts. Since our video was recorded under 30 FPS, the window size is set to 30 frames.

Finally, the task-wise accuracy is defined specifically to address the question of how well does one person perform the entire hand-hygiene process. The task-wise accuracy includes four components: $\left(1\right)$ mis-detection seconds for *rub hands with water*. $\left(2\right)$ mis-detection seconds for *rub hands without water*. $\left(3\right)$ the existence of *touch faucet with hand* after the last H2H action. $\left(4\right)$ the existence of *apply soap* between a *rub hands with water* and a *rub hands without water* action. During the evaluation of the task-based accuracy, we applied a tolerance towards the transition between actions within our untrimmed videos. A transition often occurs between two hand-hygiene actions; for example, after rubbing hands in water, a person moves their hands towards the soap. This intermediate transitional usually lasts for only a few frames and does not materially affect the hand-hygiene quality. Thus, we tolerate these actions by not counting them in our evaluation.

#### 8.2.2. Experimental Results for Hierarchical System

Table 11 shows the frame-wise (F-acc) and window-wise accuracies (W-acc) for the three systems. The multi-modal system significantly outperforms the baseline system in the cross-scenario cases, although the baseline model designed for R1C1 actually performs better on that same scenario. On average, our multi-modal hierarchical system outperforms the baseline system by 36.42 and 38.46 percentage points on cross-scenario with respect to these two measures. This demonstrates that our system design is effective for cross-scenario open-room hand-hygiene recognition. Moreover, the ideal system demonstrates that performance improvements of 3.82 and 4.19 percentage points may be possible if manual annotation of H2H actions is possible to assist domain adaptation. This improvement is best for the cross-scenario between R1C1 and R2C2.

Next, we consider task-wise evaluation of our proposed system that uses optical flow labeling and the ideal system that uses manual annotation to perform domain adaptation. These results are shown in Table 12 and Table 13, respectively. Recall that task-based accuracy considers the *duration* of the two H2H actions and the *existence* of the two H2O actions. Comparing the Table 12b and Table 13b for the models trained using R2C1, we see good cross-scenario performance for the two H2H actions. Specifically, the error in the duration is on the order of 2–4 seconds, which is comparable to similar errors for the same scenario cases. In contrast, the systems trained using R1C1 data performs worse than the other models. Many of the errors for the ideal system are nearly identical to those for the proposed system, with the notable exception of the case of model R2C2 applied to scenario R1C1, where the ideal case dramatically decreases the duration error for the two H2H actions. Since the primary difference between these two systems is the errors made by the optical flow model to facilitate domain adaptation, this points to a potential area for improvement for our model.

## 9. Discussion

In this section, we first discuss limitations of the proposed approach, and then describe suggestions for people who may be designing real-world systems for similar scenarios.

### 9.1. Discussion on System Limitations

Every system has limitations. Ours can be clustered into three areas: characteristics of the people and the environment, camera placement, and system configuration and design.

First, while we have considered cross-scenario design for an “open-room” environment, we do not expect the current system to work in unconstrained environments where objects, sinks, faucets, and water pipes may have vastly different appearances. For example, we may need to retrain parts of the system to work well for an outdoors environment, for instance in the field of a farm where produce is being harvested. Such an environment could have background vegetation moving with the wind, which could create a challenge. Variations in the appearance of the participants are also likely to require our system to be retrained. Different clothing may challenge the system, as could varied skin tone. However, we believe both of these are minor challenges because our system relies on OpenPose for locating people and their body parts, and OpenPose is relatively robust against these variations. Moreover, our system can be easily adapted to incorporate any new advances in localizing skeletal joints.

We chose to place our camera to create a side-view of the washing area. In general, the side-angle view may not be possible given the layout of the room and the position of the sink. For example, in our previous work [3], due to the size of the restroom, the side-view camera could not be placed far enough from the sink to view the entire person, or even their entire torso. In general, the camera placement may have to be adjusted to each room, so the distance to the sink and the height of the camera relative to the sink may vary. Moreover, a side-camera is vulnerable to occlusions from passing people, and depending on its distance to the sink it may not be able to observe fine motor actions of the fingers and hands. However, a side-view camera has the capability to observe other food-handling activities, which provides advantages relative to a top-down view of the sink.

With respect to our overall system, we chose a hierarchical design. This allows each sub-system to use a modality that performs well. However, if significantly more data were available, it could be possible to obtain better performance using an end-to-end model that allows all sub-systems to be integrated and trained jointly. Moreover, new advances in deep learning like the Vision Transformer [76] are producing classifiers that outperform the ResNet model we applied here.

Additionally, the domain adaptation approaches that we applied here have limitations; they are effective when there are sufficient similarities between the domains used for the design and for the deployment, but are unlikely to be effective when there are too few similarities between domains. More data and further explorations would be necessary to validate how well these methods work in a broader set of environments.

### 9.2. Suggestions for Designing Video Analytics Systems for Real-World Applications

Here we discuss some general suggestions for designing an action detection system for a real-world application and give examples from our hand-washing system throughout. Given that the variety of action detection problems is unconstrained, it is impossible to provide comprehensive design suggestions here. However, our overarching advice is to start simple and adapt from there. This may involve adding complexity if necessary, but may also require simplifying the problem even further.

For many applications, data have already been gathered and published. In these cases, it may be effective to build on their existing data and apply solutions already proposed in the literature. However, the collected data may not actually address the specific problem of interest, or no data may exist. In these cases, it is necessary to consider the data collection as an integral part of the system design.

Camera placement is a critical consideration for gathering new data for system design and evaluation. Above, we discuss several issues regarding camera placement, including being able to observe the actions of interest, being able to obtain sufficient cues about the location of the person executing the actions, and the ability of the camera to see necessary details of the actions. These must all be considered when designing a system for a real-world application. The very act of obtaining manual labels can help assess effective camera placement. If a person doing the labeling cannot see the actions or distinguish among them, then new data or a new problem definition (regarding which actions to detect) would be required.

Additional decisions about camera placement are related to ease of use for the participants. This includes the intrusiveness of wearing a camera, or implicit constraints on how people must execute actions. For example, we chose to passively observe people washing their hands which means they may not execute all steps and some actions may be out of camera view. In contrast, other handwashing systems have been designed to be interactive or even educational about what the next action to execute should be [4,5]. The interactive approach simplifies the image processing but constrains people to keep their hands over the sink in camera view during the washing activities. If a wearable head-mounted camera was chosen, people would also need to watch their hands while washing to ensure the actions are captured, and the camera itself might be intrusive.

In terms of algorithm design, it is worthwhile to consider possible domain knowledge that can be incorporated into the solution. Sometimes domain knowledge is very specific, as it would be in the case of a system that analyzes martial arts actions. In other cases, domain knowledge can be very basic, and include things that even a toddler might already know. Where’s the person? What objects do they touch and interact with? Where are those objects located? Are there other objects that should be ignored? Is there some area of the image that should be attended to or could be ignored?

In addition, we reiterate the three strategies we applied in this paper, as described in Section 2.2. Choose a common set of actions, select an ROI (either with hard attention as we did here, or training for soft attention), and use multiple modalities as needed. Related to this, consider potential hand-to-object and object-to-object interactions. These can both be informed by the domain knowledge considered above.

Finally, remember that simplifications can be defined multiple ways, including “curating the environment” (making it clean and distraction free), simplifying the set of actions to process, educating the participants to perform actions in a prescribed canonical way, in a prescribed order, and isolated from each other in time, or even choosing to do action recognition instead of action detection.

## 10. Conclusions and Future Work

In this paper, we present an overall video analytics system to solve a real-world practical problem, namely, assessing the quality of hand-hygiene actions in an open-room environment. We demonstrate that a baseline model, using a generic action-recognition CNN model performs acceptably, but only when operating in the same environment for which it was trained. We explore potential reasons for the poor performance of this model when deployed in another open-room environment, and conclude that appearance information, which is captured by the RGB inputs, is too varied and complicated, too dependent on the environment, to provide a robust feature representation for the CNN model.

Using these insights, we explore multiple modalities and seek to identify the strength of each modality. For example, given our camera placements, the skeletal modality is useful to distinguish hand-hygiene actions from non-hygiene actions, but is not adequate to distinguish among the hand-hygiene actions. By integrating multiple modalities into our final system, we are able to leverage the data, models, and approaches that have been gathered by other researchers to solve different computer vision challenges.

In our future work, we will extend this work to consider the broader class of actions associated with food handling; this includes hand hygiene, washing produce, and sanitizing containers and surfaces. We are also interested in identifying a more robust camera view for both hand-hygiene and food handling action recognition. In our previous work [3,7], the egocentric camera views from the chest and the nose share a common advantage for capturing subtle motion details in all the hand-hygiene actions. Therefore, if a food handling task involves subtle motions or relies on textural details of objects, an egocentric camera view is a good selection. However, there are also significant shortcomings of egocentric cameras; they do not always capture the action of interest, and they can be uncomfortable to wear for a long time. In contrast, static third-person cameras enable data collection to take place seamlessly without placing addition burdens on the food handler.

## Figures and Tables

**Table 1:** Actions considered, including their categorizations for this study. Note some action categorizations were different in [3,7].

Action Name	Hand-Hygiene	Hand-to-Hand	Hand-to-Object
Touch faucet with hand	Y	N	Y
Rub Hands with Water	Y	Y	N
Apply Soap	Y	N	Y
Rub Hands without Water	Y	Y	N
Swing Hands	N	-	-
Grab Paper Towel	N	-	-
Dry Hands with Paper Towel	N	-	-
Camera Occlusion	N	-	-

**Table 2:** List of regions of interest (ROI). The hand masks were found using an algorithm; all others were hand-labeled once per camera placement.

ROI	Purpose
Sink	To eliminate people not performing hand hygiene
Hands	Necessary for all hand-hygiene actions
Sanitizer	Only for the hidden-patch experiment in Section 6.2
Faucet	To assist the hand-mask model in detecting H2O actions
Soap head	To assist the hand-mask model in detecting H2O actions
Water spout	To assist the hand-mask model in detecting H2O actions, and to create waterflow ROI
Water flow	To assist distinguishing rubbing with and without water; for model training only; derived from water spout ROI

**Table 3:** Number of trimmed video clips for each scenario. Outside of the parentheses is the number of hand-hygiene actions; inside the parentheses is the number of both hand-hygiene and non-hygiene actions.

Scene	Train		Validation		Test	
Room1 cam1	127	(177)	16	(26)	60	(80)
Room2 cam1	55	(78)	9	(12)	34	(48)
Room2 cam2	96	(114)	14	(17)	53	(65)

**Table 4:** Model accuracy for all three scenarios, on the four hand-hygiene actions. **R1C1**: Room1 Camera1. **R2C1**: Room2 Camera1. **R2C2**: Room2 Camera2. **ALL**: Accuracy of total correct video prediction among all three scenarios. **H**: models only consider hand-hygiene actions. **H + N**: models consider both hand-hygiene and non-hygiene actions.

	Scene	R1C1	R2C1	R2C2	ALL
Model					
ResNet50 (H)		91.7%	97.1%	98.1%	95.2%
ResNet50 + LSTM (H)		91.8%	94.6%	93.4%	95.2%
ResNet50 + TRN (H)		93.7%	92.7%	91.4%	95.9%
ResNet50 (H + N)		92.5%	91.7%	95.4%	93.3%

**Table 5:** Model accuracy for all three scenarios on the four hand-hygiene actions. Bold numbers indicate same-scenario situation; not bolded numbers indicate where testing data is not from the same scenario as the training data. **R1C1**: Room1 Camera1. **R2C1**: Room2 Camera1. **R2C2**: Room2 Camera2. **CNN**: ResNet50. **LSTM**: ResNet50 with LSTM. **TRN**: ResNet50 with TRN. All models consider only the hand-hygiene actions.

	Scene	R1C1	R2C1	R2C2
Model				
R1C1 CNN		**91.7%**	5.9%	30.2%
R2C1 CNN		33.3%	**97.1%**	43.4%
R2C2 CNN		56.7%	73.5%	**98.1%**
R1C1 LSTM		**91.8%**	11.8%	43.4%
R2C1 LSTM		28.3%	**94.6%**	47.2%
R2C2 LSTM		35.0%	55.9%	**93.4%**
R1C1 TRN		**93.7%**	38.2%	54.7%
R2C1 TRN		58.3%	**92.7%**	45.3%
R2C2 TRN		50.0%	61.8%	**91.4%**

**Table 6:** Prediction score (no softmax) on testset, rub hands with water videos; Bold numbers indicate highest values in column; **R1C1**: Room1 Camera1. **R2C1**: Room2 Camera1. **R2C2**: Room2 Camera2; **origin**: test on regular image. **hide**: test on image with hide patch. **Act1**: touch faucet with hand. **Act2**: rub hands with water. **Act3**: rub hands without water. **Act4**: apply soap.

	Action	Act1	Act2	Act3	Act4
Model					
R1C1 origin		−1.93	**4.71**	0.02	−2.86
R1C1 hide		−1.24	**2.39**	−1.24	0.08
R2C1 origin		−1.98	**4.99**	−0.05	−3.10
R2C1 hide		−1.67	**4.44**	0.25	−3.24

**Table 7:** Upper-body skeleton detection rate for all three scenarios. **R1C1**: Room1 Camera1. **R2C1**: Room2 Camera1. **R2C2**: Room2 Camera2. **L**: left side. **R**: right side. **S**: shoulder. **E**: elbow. **W**: wrist. **H**: hand.

**(a) Left side joint detection rate.**					
	**Scene**	**Shoulder**	**Elbow**	**Wrist**	**Hand**
**Model**					
R1C1		98.4%	97.0%	91.2%	91.2%
R2C1		98.8%	98.6%	97.9%	97.9%
R2C2		98.7%	83.9%	52.7%	52.7%
**(b) Right side joint detection rate.**					
	**Scene**	**Shoulder**	**Elbow**	**Wrist**	**Hand**
**Model**					
R1C1		99.4%	99.3%	99.0%	99.0%
R2C1		95.8%	94.0%	56.4%	56.4%
R2C2		99.3%	99.2%	99.1%	99.1%

**Table 8:** ResNet50 Model accuracy with optical flow input, for all three scenarios cross recognition, to decide if an action is H2H or H2O. **R1C1**: Room1 Camera1. **R2C1**: Room2 Camera1. **R2C2**: Room2 Camera2. **flow**.

	Scene	R1C1	R2C1	R2C2
Model				
R1C1 flow		98.3%	94.1%	98.1%
R2C1 flow		95.0%	100.0%	96.2%
R2C2 flow		96.7%	94.1%	100.0%

**Table 9:** Two action average accuracy for cross scenarios only **Faucet hand**: average performance of “touch faucet with hand action”. **Soap**: average performance of “apply soap” action. **R1C1 (C)**: Model trained on Room1 Camera1, tested on other scenarios beside Room1 Camera1. **R2C1 (C)**: Model trained on Room2 Camera1, tested on other scenarios beside Room2 Camera1. **R2C2 (C)**: Model trained on Room2 Camera2, tested on other scenarios besides Room2 Camera2.

	Scene	R1C1(C)	R1C2(C)	R2C2(C)
Action				
Faucet hand		100.0%	100.0%	68.0%
Soap		90.9%	28.6%	75.0%

**Table 10:** Model accuracy for all three scenarios’ cross recognition, for the two H2H actions. **R1C1**: room1 camera1. **R2C1**: room2 camera1. **R2C2**: room2 camera2. **W**: waterflow ROI. **D**: offline data augmentation. **A**: adversarial. **I**: ideal.

	Scene	R1C1	R2C1	R2C2
Model				
R1C1 W		100.0%	88.9%	62.2%
R1C1 W + D		100.0%	96.3%	70.3%
R1C1 W + D + A		100.0%	92.6%	56.8%
R1C1 W + D + A(I)		100.0%	88.9%	81.1%
R2C1 W		73.1%	100.0%	62.2%
R2C1 W + D		84.6%	100.0%	78.4%
R2C1 W + D + A		100.0%	100.0%	83.8%
R2C1 W + D + A(I)		100.0%	100.0%	94.6%
R2C2 W		88.5%	100.0%	100.0%
R2C2 W + A		88.5%	100.0%	100.0%
R2C2 W + A(I)		100.0%	100.0%	100.0%

**Table 11:** Frame-wise accuracy (F-acc) and window-wise accuracy (W-acc) for all three scenarios, action detection. **R1C1**: room1 camera1. **R2C1**: room2 camera1. **R2C2**: room2 camera2.

**(a) Baseline system**				
	**Model**	**R1C1**	**R2C1**	**R2C2**
**Metric**				
R1C1 F-acc		85.8%	10.9%	12.3%
R1C1 W-acc		86.3%	10.3%	12.3%
R2C1 F-acc		9.7%	74.8%	34.1%
R2C1 W-acc		9.5%	79.2%	33.4%
R2C2 F-acc		38.0%	52.7%	82.8%
R2C2 W-acc		38.9%	55.5%	85.4%
**(b) Multi-modal hierarchical system**				
	**Model**	**R1C1**	**R2C1**	**R2C2**
**Metric**				
R1C1 F-acc		74.9%	67.6%	54.6%
R1C1 W-acc		76.7%	67.7%	56.7%
R2C1 F-acc		58.1%	75.1%	68.7%
R2C1 W-acc		63.1%	76.1%	71.5%
R2C2 F-acc		53.2%	74.1%	82.3%
R2C2 W-acc		57.2%	74.6%	86.1%
**(c) Ideal multi-modal hierarchical system**				
	**Model**	**R1C1**	**R2C1**	**R2C2**
**Metric**				
R1C1 F-acc		74.9%	67.1%	66.0%
R1C1 W-acc		76.7%	66.9%	69.9%
R2C1 F-acc		58.5%	75.1%	69.4%
R2C1 W-acc		63.8%	76.1%	72.8%
R2C2 F-acc		62.8%	75.3%	82.3%
R2C2 W-acc		66.7%	75.7%	86.1%

**Table 12:** Task-wise accuracy (T-acc) for all three scenarios, action detection, optical flow labeling. **R1C1**: room1 camera1. **R2C1**: room2 camera1. **R2C2**: room2 camera2. **RW**: rub hands with water. **RNW**: rub hands without water. **Faucet**: touch faucet with hand. **Soap**: apply soap.

**(a) Flow labeling: R1C1 model**					
	**Task**	**RW**	**RNW**	**Faucet**	**Soap**
**Scene**					
R1C1		0.89 s	4.11 s	67%	89%
R2C1		3.90 s	6.30 s	70%	80%
R2C2		6.87 s	8.93 s	80%	87%
**(b) Flow labeling: R2C1 model**					
	**Task**	**RW**	**RNW**	**Faucet**	**Soap**
**Scene**					
R1C1		2.67 s	3.44 s	67%	100%
R2C1		2.30 s	2.40 s	60%	90%
R2C2		4.00 s	3.80 s	27%	93%
**(c) Flow labeling: R2C2 model**					
	**Task**	**RW**	**RNW**	**Faucet**	**Soap**
**Scene**					
R1C1		6.33 s	6.11 s	89%	100%
R2C1		2.10 s	3.60 s	60%	70%
R2C2		1.60 s	2.20 s	33%	100%

**Table 13:** Task-wise accuracy (T-acc) for all three scenarios, action detection, ideal labeling. **R1C1**: room1 camera1. **R2C1**: room2 camera1. **R2C2**: room2 camera2. **RW**: rub hands with water. **RNW**: rub hands without water. **Faucet**: touch faucet with hand. **Soap**: apply soap.

**(a) Ideal labeling: R1C1 model**					
	**Task**	**RW**	**RNW**	**Faucet**	**Soap**
**Scene**					
R1C1		0.89 s	4.11 s	67%	89%
R2C1		4.00 s	6.50 s	70%	80%
R2C2		4.60 s	6.80 s	80%	93%
**(b) Ideal labeling: R2C1 model**					
	**Task**	**RW**	**RNW**	**Faucet**	**Soap**
**Scene**					
R1C1		2.00 s	2.89 s	67%	100%
R2C1		2.30 s	2.40 s	60%	90%
R2C2		3.60 s	3.67 s	27%	93%
**(c) Ideal labeling: R2C2 model**					
	**Task**	**RW**	**RNW**	**Faucet**	**Soap**
**Scene**					
R1C1		2.00 s	3.89 s	89%	89%
R2C1		1.90 s	3.20 s	60%	80%
R2C2		1.60 s	2.20 s	33%	100%

## Data Availability

The data are not publicly available because not all participants granted permission to release their associated images.
